# The Inflammatory Nexus of Bronchopulmonary Dysplasia: From Molecular Pathways to Precision Therapeutics

**DOI:** 10.1155/mi/9480568

**Published:** 2026-04-08

**Authors:** Fei Wang, Heng Zhang, Ou Jiang, Hongying Mi

**Affiliations:** ^1^ Department of Pediatrics, The First People’s Hospital of Yunnan Province, Kunming, 650032, Yunnan Province, China, ypfph.com; ^2^ School of Medicine, Kunming University of Science and Technology, 68 Wenchang Road, Kunming, 650093, Yunnan Province, China, kmust.edu.cn; ^3^ Department of Neonatology, The First People’s Hospital of Yunnan Province, Kunming University of Science and Technology Affiliated Hospital, Kunming, China, ypfph.com

**Keywords:** alveolar simplification, anti-inflammatory therapy, bronchopulmonary dysplasia, cytokines, inflammation, lung development, mesenchymal stem cell therapy, oxidative stress, premature infants, vascular dysregulation

## Abstract

Bronchopulmonary dysplasia (BPD) is a common and serious complication among preterm infants, particularly those born at extremely low gestational ages. It is primarily characterized by impaired alveolar and vascular development. Inflammation is increasingly recognized as a central mechanism in its pathogenesis. Both prenatal factors, such as intrauterine infection, and postnatal insults, including mechanical ventilation, oxygen toxicity, and infection, can trigger and sustain a dysregulated inflammatory response in the immature lung. This response involves the activation of inflammatory cells, such as neutrophils and macrophages, and the release of pro‐inflammatory mediators, reactive oxygen species (ROS), and proteases. These factors disrupt critical developmental signaling pathways and contribute to alveolar simplification and abnormal vascular growth, which are the hallmark features of BPD. Current therapeutic strategies aim to limit these inflammatory processes and support lung development. Established interventions like caffeine and corticosteroids have demonstrated varying levels of effectiveness and safety. Emerging therapies—including anti‐cytokine agents, inflammasome inhibitors, and stem cell‐based approaches—offer promising avenues by specifically targeting the inflammatory cascade. Additionally, supportive strategies such as non‐invasive ventilation, careful oxygen titration, and optimal nutrition play essential roles in reducing initial injury and facilitating recovery. Inflammation is a key mediator linking diverse perinatal insults to the disrupted lung development seen in BPD. A deeper understanding of the inflammatory mechanisms and timely, targeted interventions may offer improved outcomes for this vulnerable population.

## 1. Background

### 1.1. Bronchopulmonary Dysplasia (BPD): A Persistent Challenge in Prematurity

BPD stands as the most prevalent form of chronic lung disease affecting infants born prematurely [1–3]. It represents significant respiratory morbidity, particularly impacting infants born at extremely preterm gestational ages (less than 28 weeks) or those with extremely low birth weight (ELBW, less than 1000 g) [4]. The condition affects a substantial portion of this vulnerable population, with incidence rates reported between 30% and 50% or higher in extremely preterm cohorts [5–7]. In the United States alone, this translates to an estimated 10,000 to 15,000 new cases diagnosed each year, highlighting its public health significance [8, 9].

The impact of BPD extends far beyond the neonatal period, imposing considerable short‐term and long‐term burdens. Infants with BPD experience increased mortality rates, require prolonged hospitalizations often involving intensive care, and incur substantial healthcare costs [10–12]. Furthermore, BPD is associated with a spectrum of long‐term complications that can persist throughout childhood and into adulthood. These include recurrent respiratory infections, an increased propensity for reactive airway disease or asthma, diminished exercise tolerance, and the development of pulmonary hypertension (PH) [13, 14]. Systemic consequences such as systemic hypertension, growth delays, and adverse neurodevelopmental outcomes, including delays in motor skills and language development, are also more common in infants with BPD [13, 15]. The persistence of abnormal lung function and structure into adulthood raises concerns about increased susceptibility to chronic obstructive pulmonary disease (COPD) later in life [10, 16]. The enduring nature of these sequelae firmly places BPD within the framework of the “Developmental Origins of Health and Disease” (DOHaD) concept, where early life events significantly influence long‐term health trajectories [2].

### 1.2. The Evolving Phenotype: From “Old” to “New” BPD

The clinical and pathological understanding of BPD has evolved considerably since its initial description by Northway and colleagues in 1967 [5, 17, 18]. The “classic” or “old” BPD primarily affected relatively more mature preterm infants (e.g., mean gestation of 34 weeks PMA in Northway’s cohort 5) who had survived severe hyaline membrane disease, now known as respiratory distress syndrome (RDS), following treatment with high concentrations of supplemental oxygen and aggressive positive pressure mechanical ventilation [19, 20]. Histopathologically, this form was characterized by significant airway injury, marked inflammation, widespread interstitial fibrosis, and emphysematous or cystic changes in the lung parenchyma [2, 21].

Over the past few decades, significant advancements in perinatal and neonatal care have dramatically altered the landscape of BPD [8, 22, 23]. The widespread adoption of antenatal corticosteroids to promote fetal lung maturation, postnatal surfactant replacement therapy to treat RDS, and the implementation of gentler ventilation strategies, including non‐invasive support like continuous positive airway pressure (CPAP), have markedly improved the survival rates of extremely premature infants [2, 5, 24]. Paradoxically, while these advances have reduced the incidence of classic BPD in more mature preterm infants [8, 25, 26], the increased survival of infants born at the edge of viability has led to the emergence of a different phenotype, often termed “new” BPD [8, 27–29].

This “new” BPD predominantly affects infants born at very early gestational ages, typically during the late canalicular or early saccular stages of lung development (often <28−30 weeks gestation) [5, 30, 31]. Pathologically, it is defined as an arrest in lung development rather than severe destructive injury and fibrosis [2, 32, 33]. The key features are impaired alveolar septation, resulting in fewer, larger, and structurally simplified alveoli (alveolar simplification), and dysregulated development of the pulmonary vasculature, leading to abnormal capillary formation and distribution and vascular remodeling [8, 34]. While inflammation remains a crucial component of the pathogenesis of “new” BPD, overt fibrosis and cystic changes are typically less prominent compared to the classic form [2, 34].

### 1.3. Inflammation: A Central Mediator of BPD Pathogenesis

A substantial and growing body of evidence derived from clinical observations, biomarker studies in human infants, and various animal models consistently points towards inflammation as a major contributor to, and a common denominator in, the complex, multifactorial pathogenesis of BPD [13, 35]. BPD is increasingly conceptualized as the outcome of an imbalance between injurious stimuli acting on the developing lung and the lung’s intrinsic capacity for repair and maturation [8, 36]. Persistent, unresolved inflammation appears to be a key factor that tips this balance towards injury and disrupts the normal programed developmental trajectory, representing an aberrant or maladaptive reparative response [37].

This detrimental inflammatory process is not initiated by a single cause but rather by a confluence of factors, often referred to as “multiple hits,” acting upon a susceptible, structurally and immunologically immature lung [38, 39]. These hits include prenatal exposures, most notably chorioamnionitis (intra‐amniotic infection and/or inflammation), and a range of postnatal insults encountered in the neonatal intensive care unit (NICU), such as the need for mechanical ventilation, exposure to supplemental oxygen (hyperoxia), and the occurrence of postnatal infections or sepsis [39, 40]. The interplay between these factors and the infant’s genetic predisposition ultimately determines the likelihood and severity of BPD development [38].

However, it is critical to acknowledge that inflammation, while central, is likely not the sole driver of BPD pathology [41]. The clinical presentation of BPD is highly heterogeneous, suggesting the existence of distinct endotypes—some driven predominantly by inflammation, while others may stem primarily from vascular dysregulation or growth arrest unrelated to immune activation [42]. Furthermore, potent anti‐inflammatory agents like glucocorticoids can paradoxically induce alveolar simplification, indicating that simply suppressing inflammation does not guarantee normal lung development. Thus, inflammation should be viewed as a critical, but not exclusive, component of a complex developmental disruption [43].

## 2. BPD: Definition, Pathophysiology, and Diagnosis

### 2.1. Defining BPD: An Evolving Challenge

Establishing a precise and universally accepted definition for BPD has been a persistent challenge since Northway’s seminal description in 1967 [5]. The definition has necessarily evolved over time to reflect significant changes in the clinical presentation of the disease, the characteristics of the affected infant population (increasing survival of extremely premature infants), and advancements in neonatal respiratory care [5, 8].

In 2001, a workshop convened by the National Institute of Child Health and Human Development (NICHD) proposed a consensus definition that gained widespread acceptance and remains commonly used [13]. This definition applies primarily to infants born at less than 32 weeks gestation. It defines BPD based on the requirement for supplemental oxygen (fraction of inspired oxygen, FiO_2_ > 0.21) for at least 28 consecutive days [13, 44]. The assessment of BPD presence and severity is then made at 36 weeks postmenstrual age (PMA) or discharge to home, whichever comes first. Severity is graded based on the level of respiratory support needed at the time of assessment:

a. **Mild BPD:** Breathing room air (FiO_2_ = 0.21) at 36 weeks PMA.
b. **Moderate BPD:** Needing supplemental oxygen (FiO_2_ < 0.30) at 36 weeks PMA.
c. **Severe BPD:** Needing supplemental oxygen (FiO_2_ ≥ 0.30) and/or positive pressure support (invasive or non‐invasive) at 36 weeks PMA [13, 45].

A subsequent NICHD workshop in 2018 proposed modifications to the severity grading, focusing on the mode of respiratory support at 36 weeks PMA, irrespective of FiO_2_ for non‐invasive support [13, 46]. This revised grading classifies severity as:

a. **Grade 1 BPD:** Receiving nasal cannula flow ≤ 2 L/min.
b. **Grade 2 BPD:** Receiving nasal cannula flow > 2 L/min or non‐invasive positive pressure ventilation (NIPPV).
c. **Grade 3 BPD:** Receiving invasive mechanical ventilation [13].

Preliminary evidence suggests this support‐based grading system may better predict respiratory morbidity in early childhood compared to the 2001 FiO_2_‐based severity scale [13, 46].

### 2.2. Pathophysiology: Arrested Lung Development

As previously discussed, the fundamental pathophysiology of contemporary (“new”) BPD is understood as a disruption or arrest of normal lung development occurring during a critical window, specifically the late canalicular and saccular stages (roughly corresponding to 24–36 weeks of gestation) [25]. This developmental arrest manifests primarily in two interconnected structural abnormalities: alveolar simplification and dysregulated pulmonary vascular growth (Figure 1).

Figure 1Comparison of pathological features in “classic” versus “new” bronchopulmonary dysplasia (BPD). (A) Classic BPD’ (pre‐surfactant era) characterized by marked airway injury with epithelial metaplasia (black arrows), prominent inflammation with inflammatory cell infiltration (yellow arrows), widespread interstitial fibrosis (blue arrows), and emphysematous or cystic changes (asterisks). (B) New BPD’ (post‐surfactant era) primarily defined by developmental arrest with alveolar simplification—fewer, larger, and structurally simplified alveoli (black arrows) and dysregulated pulmonary vascular development with decreased microvascular density and abnormal vessel distribution (red arrows). Note the relative absence of fibrosis and cystic changes in new BPD compared to the classic form.

**Figure:**
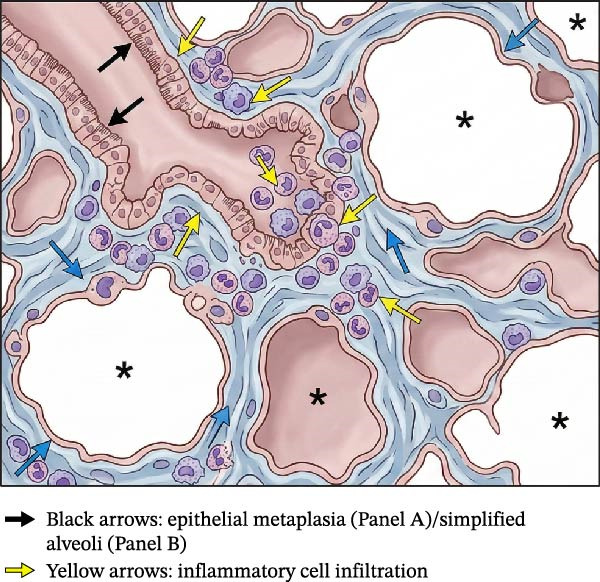
(A)

**Figure:**
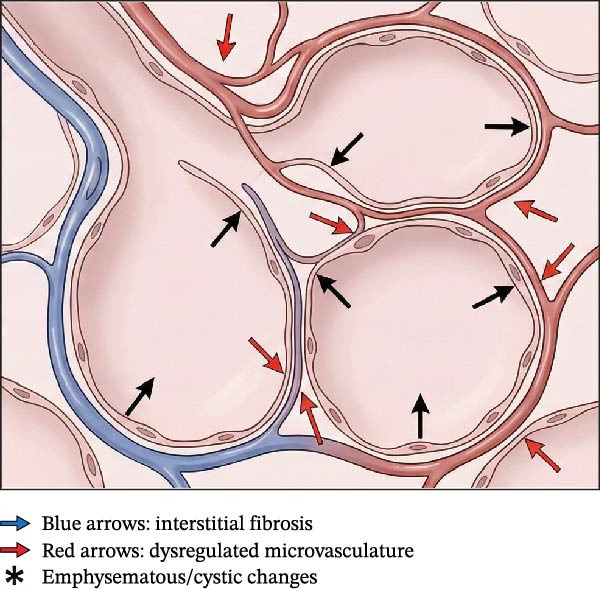
(B)

#### 2.2.1. Alveolar Simplification

This is considered the histological hallmark of new BPD [8]. It results from the failure of secondary septation, the complex process by which the terminal saccules subdivide to form the vast number of mature alveoli necessary for efficient gas exchange [40, 47]. In BPD, this process is inhibited or arrested, leading to lungs with significantly fewer alveoli that are abnormally large and simplified in structure [4, 8]. This reduction in alveolar number and the consequent decrease in the total alveolar surface area available for gas exchange are major contributors to the respiratory insufficiency seen in affected infants [48].

#### 2.2.2. Dysregulated Vascular Development

Occurring in parallel with impaired alveolarization is a significant disruption of pulmonary vascular growth and structure, often termed “dysmorphic pulmonary vascularization” or “dysregulated angiogenesis” [8]. Normal lung development involves tightly coordinated growth of the airways and the pulmonary vasculature to ensure adequate perfusion of the gas‐exchanging units. In BPD, this coordination is lost [49]. Pathological features include a marked reduction in the number of small pulmonary blood vessels and capillaries within the alveolar walls (decreased microvascular density), an abnormal distribution of the remaining vessels, and structural remodeling of pulmonary arterioles [48, 50]. This remodeling often involves hypertrophy and hyperplasia of smooth muscle cells in the vessel walls, leading to thickened, less compliant arterioles [51]. This abnormal vascular development not only further compromises gas exchange efficiency by creating ventilation‐perfusion mismatch but also significantly increases pulmonary vascular resistance (PVR) [49, 52]. Elevated PVR is the primary driver for the development of PH, a frequent and serious complication associated with increased morbidity and mortality in infants with BPD [10, 53].

### 2.3. Diagnosis and Evaluation

The diagnosis of BPD currently remains a clinical one, integrating information about the infant’s gestational age at birth, birth weight, clinical history (particularly the need for respiratory support for prematurity‐related lung disease like RDS), and the duration and type of respiratory support required postnatally [5, 54]. The most commonly applied diagnostic framework is the NICHD 2001 criteria or its 2018 modification, which relies on the assessment of respiratory support needs at 36 weeks PMA [13, 55].

Ongoing evaluation of infants diagnosed with or at high risk for BPD involves meticulous monitoring of their respiratory status. This includes tracking oxygen requirements (FiO_2_), assessing the work of breathing, monitoring oxygen saturation levels via continuous pulse oximetry, and performing arterial or capillary blood gas analyses to assess for hypoxia, hypercarbia, or acidosis [56]. Chest radiography is commonly performed; typical findings in established BPD can include diffuse haziness, low lung volumes, linear opacities, and in more severe or classic forms, cystic changes or hyperinflation [57]. However, radiographic findings in “new” BPD can be less specific, often showing relatively clear lungs despite significant gas exchange impairment, reflecting the underlying pathology of alveolar simplification rather than gross fibrosis or destruction [58].

While clinical criteria remain the mainstay of diagnosis, research continues to explore the potential utility of biomarkers for earlier prediction or more objective assessment of BPD severity [2, 59]. Potential biomarkers include inflammatory mediators measured in tracheal aspirate fluid or blood (e.g., cytokines like IL‐6, and IL‐8 [38], markers of lung injury or repair, genetic markers, or distinct metabolic profiles identified through metabolomic analyses [13]. However, none of these biomarkers have yet achieved sufficient validation for routine clinical use in diagnosing or managing BPD [13, 60].

## 3. The Inflammatory Cascade in BPD Pathogenesis

### 3.1. Inflammation as the Convergent Pathway

**Figure 2:**
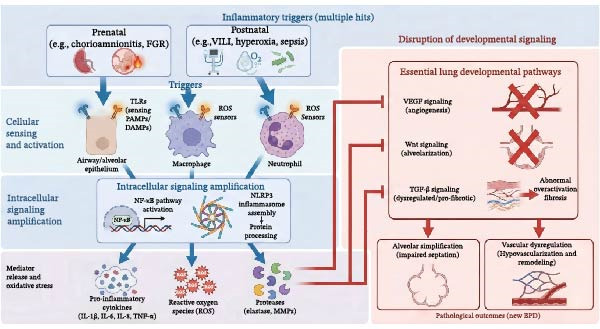
The inflammatory cascade in bronchopulmonary dysplasia pathogenesis. The schematic illustrates the “multiple hit” hypothesis where prenatal factors (e.g., chorioamnionitis, FGR) and postnatal insults (e.g., VILI, hyperoxia, sepsis) act as cumulative triggers. These insults are sensed by Toll‐like receptors (TLRs) and ROS sensors on airway/alveolar epithelial cells, macrophages, and neutrophils. This activation triggers intracellular signaling amplification via the NF‐κB pathway and NLRP3 inflammasome assembly. The consequent release of pro‐inflammatory cytokines (IL‐1β, IL‐6, IL‐8, TNF‐α), reactive oxygen species (ROS), and proteases creates a toxic microenvironment. Crucially, this inflammatory milieu actively disrupts essential lung developmental pathways: inhibiting VEGF signaling (angiogenesis) and Wnt signaling (alveolarization), while dysregulating TGF‐β signaling (promoting fibrosis). This molecular disruption leads to the hallmark pathological outcomes of “new” BPD: alveolar simplification and vascular dysregulation.

The pathogenesis of BPD is recognized as multifactorial, arising from a complex interplay between the intrinsic vulnerability of the immature lung, genetic predisposition, and exposure to various injurious stimuli both before and after birth [13, 61]. Amidst this complexity, inflammation has emerged as a critical convergent pathway, representing a common biological response that links diverse etiological factors to the ultimate outcome of disrupted lung development (Figure 2) [4]. The resulting inflammatory cascade, whether initiated by intrauterine infection, mechanical stretch from ventilation, oxygen toxicity, or postnatal sepsis, is a key driver of the lung injury and aberrant repair processes characterizing BPD [62].

A defining feature of the inflammatory response in the context of BPD is its often exaggerated and persistent nature [48]. Unlike a typical, self‐limited inflammatory response that resolves following the removal of the inciting stimulus, the inflammation in the developing lungs of preterm infants prone to BPD frequently fails to resolve appropriately [63]. This sustained inflammation leads to ongoing tissue damage, creates a microenvironment unfavorable for normal developmental processes, and actively interferes with the critical signaling pathways that orchestrate alveolar and vascular growth [8].

### 3.2. Triggers of Inflammation

The inflammatory cascade in BPD is initiated by a “multiple hit” process. As summarized in Table 1, these triggers span the prenatal and postnatal periods. Prenatal factors such as chorioamnionitis and fetal growth restriction (FGR) prime the immature lung, rendering it susceptible to subsequent injuries. Postnatally, mechanical ventilation (VILI), oxygen toxicity (hyperoxia), and sepsis act as cumulative insults. These factors collectively induce epithelial and endothelial damage, recruiting inflammatory cells and perpetuating a cycle of injury that disrupts alveolarization.

**Table 1:** Key inflammatory components and triggers in BPD pathogenesis.

Category	Specific element	Key role in BPD	Key supporting references
Triggers			
Prenatal	Chorioamnionitis/Intra‐amniotic Infection	Initiates fetal lung inflammation, FIRS; potential priming for postnatal injury	[38]
	Fetal Growth Restriction (FGR)/IUGR	Associated with placental insufficiency, hypoxia, and potential inflammation; linked to vascular maldevelopment	[48]
Postnatal	Mechanical ventilation (VILI)	Baro/volu/atele/biotrauma; causes mechanical stress, epithelial/endothelial injury, triggers inflammation	[38]
	Hyperoxia (oxygen toxicity)	Generates excessive ROS, causes oxidative stress, directs cell damage, activates inflammatory signaling	[38]
	Infection/Sepsis	Amplifies pulmonary and systemic inflammation and exacerbates lung injury	[38]
	Necrotizing Enterocolitis (NEC)	Triggers systemic inflammatory response (SIRS) linked to BPD risk	[13]
	Patent Ductus Arteriosus (PDA)	Pulmonary over circulation, edema, potential contribution to injury/inflammation	[40]
Cell types			
Innate immune	Neutrophils (PMNs)	Early responders; release proteases (elastase), ROS, and pro‐inflammatory cytokines; contributing to acute tissue damage	[40]
	Macrophages (Alveolar/Interstitial)	Phagocytosis; M1 phenotype (pro‐inflammatory cytokine release, ROS); M2 phenotype (resolution, repair); imbalance contributes to chronic inflammation	[40]
Mediator types			
Cytokines	IL‐1β, IL‐6, IL‐8 (CXCL8), TNF‐α	Key pro‐inflammatory signals; recruit/activate immune cells; and mediate tissue damage; elevated levels are biomarkers for BPD risk	[40]
Chemokines	IL‐8 (CXCL8), others	Recruit specific inflammatory cells (neutrophils, monocytes) to the lung	[38]
Reactive species	Reactive oxygen species (ROS)	Cause oxidative damage to cells/molecules; activate pro‐inflammatory signaling (e.g., NF‐κB)	[10]
Proteases	Neutrophil Elastase, Matrix Metalloproteinases (MMPs)	Degrade extracellular matrix; contribute to tissue remodeling; protease/anti‐protease imbalance implicated	[63]
Lipid mediators	Leukotrienes	Chemoattraction, increased vascular permeability, and vasoconstriction; contribute to increased PVR	[64]
Growth factors	TGF‐β, CTGF	Dysregulated signaling promotes fibrosis, inhibits alveolarization, vascular remodeling	[40]
DAMPs	HMGB1, Hyaluronan fragments (LMW HA)	Endogenous danger signals released from damaged cells; activate innate immunity (TLRs, inflammasomes), perpetuate sterile inflammation	[40]
Adipocytokines	Visfatin	Elevated levels of BPD, potentially associated with inflammation	[65]
Signaling pathways			
Transcription	NF‐κB	Master regulator activated by ROS, cytokines, TLRs; drives expression of multiple pro‐inflammatory genes	[65]
Innate sensing	Toll‐Like Receptors (TLRs; esp. TLR2, TLR4)	Recognize PAMPs (e.g., LPS) and DAMPs; trigger inflammatory signaling cascades (NF‐κB, etc.)	[40]
	Inflammasomes (e.g., NLRP3)	Sense cellular stress/damage/PAMPs/DAMPs; activate caspase‐1 leading to maturation/secretion of IL‐1β and IL‐18	[40]
Developmental	TGF‐β, Wnt, VEGF	Essential for lung development; pathways are disrupted/dysregulated by inflammation and injury, leading to arrested development	[40]

Conditions associated with placental insufficiency are significant risk factors. It is important to distinguish between Fetal Growth Restriction (FGR), which implies a pathological restriction of genetic growth potential often due to placental dysfunction, and Intrauterine Growth Restriction (IUGR), a broader term [66, 67]. FGR is strongly associated with BPD, likely mediated through shared pathways involving compromised vascular development and a heightened susceptibility to inflammatory stimuli [68–70].

### 3.3. Key Inflammatory Cells

The inflammatory infiltrate in the lungs of infants developing BPD is characterized by the accumulation of specific types of immune cells, primarily neutrophils and macrophages [40, 71].

#### 3.3.1. Neutrophils (Polymorphonuclear Leukocytes, PMNs)

These are typically the first responders recruited from the circulation to sites of injury or infection in the lung [72]. Attracted by chemotactic signals (like IL‐8), neutrophils migrate into the alveolar spaces and interstitium [73]. While essential for clearing pathogens, their activation also leads to the release of a potent arsenal of cytotoxic substances, including proteolytic enzymes (such as neutrophil elastase), reactive oxygen species (ROS) generated via the respiratory burst, and various pro‐inflammatory cytokines [8, 64]. In the context of BPD, excessive or prolonged neutrophil presence and activation contribute significantly to acute lung injury, damaging epithelial and endothelial cells and degrading the extracellular matrix [74].

#### 3.3.2. Macrophages

These phagocytic cells also accumulate significantly in the airways and lung tissue during the development of BPD [75]. Lung macrophages, particularly alveolar macrophages, play a complex and multifaceted role in the inflammatory process. They can adopt different activation states or phenotypes. Classically activated (M1) macrophages are potent pro‐inflammatory cells, stimulated by factors like LPS and interferon‐gamma. They produce high levels of pro‐inflammatory cytokines (such as TNF‐α, IL‐1β, IL‐6, and IL‐12), generate ROS and nitric oxide (NO), and contribute to pathogen killing and tissue damage [64]. Conversely, alternatively activated (M2) macrophages are typically induced by cytokines like IL‐4 and IL‐10 and are generally associated with the resolution of inflammation, tissue repair, wound healing, and immune suppression. In BPD, there may be a persistent predominance of pro‐inflammatory M1 macrophages or a failure to effectively switch towards the M2 phenotype, contributing to the chronic, non‐resolving inflammation that hinders normal lung development [62].

### 3.4. Key Inflammatory Mediators

A complex network of soluble mediators orchestrates the inflammatory response (Table 1). Pro‐inflammatory cytokines (IL‐1β, IL‐6, IL‐8, TNF‐α) are consistently elevated in BPD patients and recruit neutrophils and macrophages. ROS generated by hyperoxia and inflammatory cells cause direct oxidative damage and activate signaling pathways like NF‐κB. Additionally, an imbalance between proteases (e.g., neutrophil elastase, MMPs) and antiproteases leads to extracellular matrix degradation, while DAMPs released from damaged cells perpetuate sterile inflammation.

### 3.5. Implicated Signaling Pathways

The actions of inflammatory triggers and mediators are transduced through complex intracellular signaling pathways, which ultimately regulate gene expression, cell function, and fate. Several key pathways are implicated in BPD‐associated inflammation:

#### 3.5.1. Nuclear Factor‐Kappa B (NF‐κB)

This transcription factor pathway is a master regulator of inflammation [65]. It can be activated by a wide range of stimuli relevant to BPD, including pro‐inflammatory cytokines (TNF‐α, IL‐1β), bacterial products (LPS via TLR4), viral components, and oxidative stress (OS) [76, 77]. Activation typically involves the phosphorylation and degradation of inhibitory IκB proteins, allowing NF‐κB dimers (commonly p50/RelA) to translocate to the nucleus. There, NF‐κB binds to specific DNA sequences in the promoter regions of target genes, driving the transcription of numerous pro‐inflammatory molecules, including cytokines (TNF‐α, IL‐1, IL‐6), chemokines (IL‐8), adhesion molecules (ICAM‐1, VCAM‐1), and enzymes involved in inflammation (e.g., iNOS, COX‐2) [78].

#### 3.5.2. Toll‐Like Receptors (TLRs)

TLRs are a critical family of pattern recognition receptors (PRRs) central to the innate immune system. They recognize conserved molecular structures known as pathogen‐associated molecular patterns (PAMPs), such as LPS (recognized by TLR4) and bacterial lipoproteins (recognized by TLR2), as well as endogenous DAMPs released from damaged cells [79]. TLR signaling, particularly through TLR2 and TLR4, has been implicated in BPD pathogenesis, triggered by potential prenatal or postnatal infections or by sterile inflammation [80].

#### 3.5.3. Inflammasomes

These are intracellular multi‐protein complexes that function as sensors of cellular stress, infection, and damage. The best‐characterized is the NLRP3 inflammasome. Upon activation by diverse stimuli, including PAMPs, DAMPs (like LMW HA, ATP, uric acid crystals), ROS, and lysosomal damage, the NLRP3 inflammasome oligomerizes and recruits the adaptor protein ASC and pro‐caspase‐1. This leads to the auto‐activation of caspase‐1, which then cleaves pro‐IL‐1β and pro‐IL‐18 into their mature, biologically active forms, promoting their secretion and potent pro‐inflammatory effects [81, 82].

#### 3.5.4. Developmental Signaling Pathways (Interaction With Inflammation)

Critically, the inflammatory cascade does not operate in isolation but actively intersects with and disrupts the signaling pathways essential for normal lung development. This interference is a key mechanism by which inflammation leads to the structural abnormalities of BPD [8].

##### 3.5.4.1. Transforming Growth Factor‐Beta (TGF‐β) Signaling

This pathway plays complex roles in lung development, regulating cell proliferation, differentiation, and ECM production [38]. However, in the context of lung injury and inflammation, TGF‐β signaling is often upregulated and can become pathogenic, promoting excessive ECM deposition (fibrosis), inhibiting alveolar epithelial cell proliferation necessary for septation, and contributing to smooth muscle proliferation in airways and vessels [83]. Anti‐inflammatory agents like PPAR‐γ agonists may exert protective effects in part by modulating aberrant TGF‐β signaling [84].

##### 3.5.4.2. Wnt Signaling

The Wnt family of signaling pathways (canonical and non‐canonical) is fundamentally important for lung morphogenesis, including branching and alveolarization. Inflammation and injury can dysregulate Wnt signaling, contributing to impaired alveolar development. Aberrant Wnt signaling has been implicated in hyperoxia‐induced lung injury models, and modulation by therapies like PPAR‐γ agonists has shown promise [85].

##### 3.5.4.3. Vascular Endothelial Growth Factor (VEGF) Signaling

VEGF‐A is a master regulator of angiogenesis (new blood vessel formation), essential for the development of the pulmonary capillary network that intertwines with developing alveoli [10]. Both hyperoxia and inflammation have been shown to suppress VEGF expression or signaling in the developing lung [62, 86]. This suppression is considered a major mechanism underlying the impaired vascular growth (reduced capillary density) characteristic of BPD [87].

## 4. Inflammation‐Driven Impairment of Lung Development

### 4.1. Mechanisms of Impaired Alveolarization

The hallmark feature of “new” BPD, alveolar simplification, stems directly from the inhibitory effects of persistent inflammation on the process of secondary septation. This intricate developmental process, normally occurring during the saccular and early alveolar stages, involves the outgrowth of fibroblastic crests containing capillaries into the saccular airspaces, effectively subdividing them into smaller, more numerous alveoli. Inflammation disrupts this process through multiple mechanisms [88].

Firstly, the inflammatory microenvironment itself is toxic to the key cellular players involved in septation [10]. Pro‐inflammatory mediators like TNF‐α and IL‐1β, along with high levels of ROS generated by hyperoxia and inflammatory cells, can directly damage or induce apoptosis in alveolar epithelial cells (both type I and type II pneumocytes) and crucial mesenchymal cells, including alveolar myofibroblasts which are thought to drive septal crest formation [88, 89]. Damage to type II cells is particularly detrimental as they serve as progenitors for type I cells and produce surfactant [90].

Secondly, inflammation profoundly dysregulates the delicate balance of growth factors and signaling pathways that normally orchestrate alveolar morphogenesis [91]. As previously noted, excessive or prolonged signaling through the TGF‐β pathway, often activated by injury, can inhibit epithelial cell proliferation and promote the differentiation of fibroblasts into myofibroblasts that deposit excessive extracellular matrix, hindering rather than promoting normal septation [92, 93]. Similarly, disruptions in Wnt signaling, another pathway crucial for alveolar development, have been implicated in inflammation‐ and hyperoxia‐induced lung injury models [85]. The normal spatial and temporal expression patterns of these and other growth factors (e.g., FGFs, PDGFs) are likely perturbed by the inflammatory milieu.

Thirdly, the balance between proteases and anti‐proteases is disrupted [63]. Excessive activity of proteases like neutrophil elastase and MMPs, released by infiltrating inflammatory cells, can degrade essential components of the extracellular matrix, such as elastin and collagen [64]. Elastin, in particular, is critically important for the recoil properties of the lung and is thought to play a key role in anchoring and guiding septal formation. Its degradation can compromise the structural scaffold required for proper alveolarization [63].

Emerging evidence highlights the role of cellular senescence—a state of irreversible growth arrest [94]. Stressors like hyperoxia and inflammation induce premature senescence in lung cells. These senescent cells secrete a senescence‐associated secretory phenotype (SASP), releasing pro‐inflammatory cytokines and proteases that propagate inflammation and hinder tissue regeneration. Targeting these cells with senolytics represents a novel, albeit experimental, therapeutic avenue that warrants further investigation to determine its safety and efficacy in the developing lung [95].

### 4.2. Mechanisms of Dysregulated Angiogenesis and Vascular Remodeling

The abnormal pulmonary vascular development characteristic of BPD is also tightly linked to inflammation [40, 96]. Normal lung development requires coordinated growth of the capillary network alongside the developing airways and alveoli (angiogenesis) to ensure adequate gas exchange. Inflammation disrupts this process, leading to both insufficient vessel growth (hypovascularization) and structural abnormalities in the vessels that do form (remodeling).

A key mechanism underlying the reduced capillary density seen in BPD is the suppression of crucial pro‐angiogenic signaling pathways, most notably the VEGF pathway [97]. VEGF‐A is essential for stimulating endothelial cell proliferation, migration, survival, and the formation of new capillaries [98]. Both experimental hyperoxia and inflammation have been shown to decrease the expression or bioavailability of VEGF and its receptors (e.g., VEGFR2) in the developing lung [86]. This suppression of VEGF signaling directly impairs angiogenesis, contributing significantly to the paucity of alveolar capillaries observed in BPD lungs [99].

Concurrently, inflammation promotes abnormal remodeling of the existing pulmonary arterioles [100]. Pro‐inflammatory cytokines (like IL‐1β, IL‐6, and TNF‐α) and other mediators released during inflammation can directly stimulate the proliferation and migration of pulmonary artery smooth muscle cells (PASMCs) [101]. These cells then accumulate in the vessel walls, leading to medial hypertrophy (thickening). Inflammation also promotes the deposition of extracellular matrix proteins (like collagen) within the vessel walls, further contributing to thickening and stiffening [101].

Furthermore, inflammation and OS induce endothelial dysfunction, impairing the normal regulatory functions of the endothelium, such as the production of vasodilators like NO [101]. Endothelial activation and injury also contribute to a pro‐coagulant and pro‐inflammatory state within the vasculature. Activation of receptors like TLR4 on vascular cells by inflammatory stimuli (e.g., LPS, potentially saturated fatty acids) can directly mediate these inflammatory responses within the vessel wall [102].

The combination of reduced capillary density (impaired angiogenesis) and thickened, stiffened, and dysfunctional arterioles (vascular remodeling) leads to a significant increase in PVR [49]. This elevated PVR is the hemodynamic basis for the development of PH, a common and life‐threatening complication of severe BPD [10].

### 4.3. The Interplay Between OS and Inflammation

**Figure 3:**
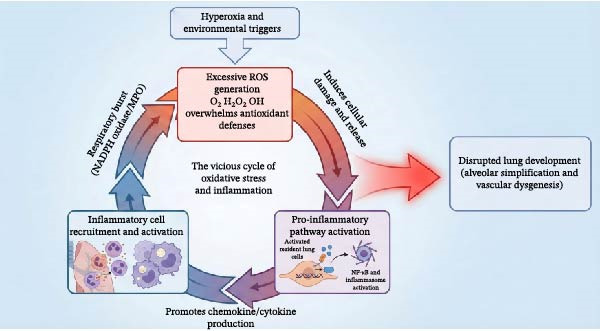
The vicious cycle of oxidative stress and inflammation in bronchopulmonary dysplasia. This diagram depicts the self‐perpetuating feedback loop that drives chronic lung injury. Hyperoxia and environmental triggers initiate the cycle by generating excessive reactive oxygen species (ROS), which overwhelm the immature antioxidant defenses. This oxidative stress induces cellular damage and releases damage‐associated molecular patterns (DAMPs), activating pro‐inflammatory pathways (NF‐κB and inflammasomes) in resident lung cells. This leads to the production of chemokines/cytokines that recruit and activate inflammatory cells (neutrophils and macrophages). These activated cells undergo a respiratory burst (mediated by enzymes like NADPH oxidase and MPO), releasing further waves of ROS, thus closing the vicious cycle. This persistent cycle continuously disrupts lung development, resulting in alveolar simplification and vascular dysgenesis.

OS and inflammation are critically linked in BPD pathogenesis, forming a detrimental positive feedback loop. Postnatal hyperoxia initiates this cycle by generating an excess of ROS that overwhelms the premature lung’s underdeveloped antioxidant defenses, leading to ROS accumulation [103]. Activated inflammatory cells then produce large quantities of their own ROS, establishing a vicious cycle where OS fuels inflammation and inflammation generates more OS. This process perpetuates injury, prevents inflammatory resolution, disrupts normal lung development signals, and ultimately drives the BPD phenotype (Figure 3).

## 5. Therapeutic Strategies Targeting Inflammation in BPD

### 5.1. Rationale for Anti‐Inflammatory Therapies

Given that inflammation acts as the linchpin linking perinatal insults to arrested lung development, modulating this response is a logical therapeutic priority. The primary goal is to dampen injurious inflammation early enough to permit normal alveolarization and angiogenesis. As summarized in Table 2, therapeutic approaches range from established pharmacological agents to emerging regenerative strategies.

**Table 2:** Overview of anti‐inflammatory and related therapeutic strategies for BPD.

Therapy/strategy	Primary mechanism(s)	Key preclinical evidence (brief)	Key clinical evidence (brief)	Current status/key considerations
Established/commonly used				
Corticosteroids (systemic; e.g., Dexa)	Broad anti‐inflammatory/immunosuppressive	Reduces inflammation, and improves lung structure in models	Reduces BPD/death (esp. later use); facilitates extubation	Effective but significant short/long‐term risks (esp. neurodevelopment with early use); use cautiously/selectively [13]
Caffeine	Respiratory stimulant; anti‐inflammatory reduces ER stress/mito dysfunction?	Reduces inflammation, and improves alveolarization in models	Reduces BPD; improves neurodevelopmental outcome (CAP trial)	Standard of care for apnea; established BPD prevention strategy with good safety profile [10]
Vitamin A (IM)	Epithelial differentiation/repair; antioxidant; anti‐inflammatory	Improves lung structure in some models	Modestly reduces BPD/death (meta‐analyses	Efficacy is established but limited use due to the IM route; the enteral route is ineffective [10]
Investigational/emerging				
Antioxidants (SOD, NAC, Vit E/C, etc.)	Scavenge ROS and boost antioxidant defenses.	Variable results in models	Generally failed to show consistent benefit in large clinical trials/meta‐analyses [10]	Largely ineffective as currently tested; challenges in delivery, timing, dose?
Inhaled nitric oxide (iNO)	Pulmonary vasodilator; anti‐inflammatory/anti‐proliferative effects	Attenuates inflammation, and improves structure in some models	Large RCTs showed mixed/no benefit for BPD prevention	Not recommended for routine BPD prevention
Vitamin D	Immunomodulation (e.g., TLR4 downregulation); promotes alveolarization.	Improves alveolarization, reduces inflammation in models	Limited clinical data for BPD prevention; association studies ongoing	Investigational; role of supplementation unclear
PPAR‐γ agonists	Anti‐inflammatory nuclear receptor activation; modulates TGF‐β/Wnt	Dampens inflammation improves alveolar/vascular structure in models	No large clinical trials in BPD yet	Promising preclinical data; clinical translation pending safety/efficacy studies
CC10 (club cell protein)	Endogenous anti‐inflammatory protein	Anti‐inflammatory effects *in vitro*	Phase I trial showed reduced airway inflammation markers	Investigational; larger efficacy trials needed
IL‐1Ra (Anakinra)	Blocks IL‐1β signaling	Reduces inflammation, and improves alveolarization in models	No large clinical trials in BPD yet	Targeted approach based on IL‐1β role; investigational
NLRP3 inflammasome inhibitors	Block activation of the inflammasome, reducing IL‐1β/IL‐18 production	Preclinical studies emerging	No clinical trials in BPD yet	Targeted approach; investigational
SP‐D (recombinant human)	Innate immunity; anti‐inflammatory (TLR4 modulation?)	Reduces lung inflammation in models	Low endogenous levels linked to BPD; no large clinical trials of rhSP‐D yet	Investigational; proposed as potential therapy [104]
Stem cells (MSCs)	Paracrine effects: anti‐inflammatory, antioxidant, pro‐angiogenic, anti‐fibrotic, immunomodulatory	Consistently improves lung structure/function, and reduces inflammation/OS in diverse models [10]	Early phase trials show feasibility/safety; efficacy data awaited from larger RCTs [10]	Promising regenerative approach; investigational, optimizing delivery/dose/source ongoing
Macrolides (e.g., Azithromycin)	Antibacterial; potential immunomodulatory effects	Limited specific BPD models	One RCT suggested benefit in *Ureaplasma* + infants; more data needed [10]	Investigational for BPD; concerns re: resistance, need more safety/efficacy data [10]
Supportive Care				
Gentle ventilation (NIV, VTV, etc.)	Minimize VILI (baro/volu/atele/biotrauma)	Reduces lung injury/inflammation vs aggressive ventilation	Associated with improved outcomes, reduced need for invasive support	Standard of care; fundamental to BPD prevention [37]
Optimized oxygen management	Avoid hyperoxia and hypoxia	Reduces ROS generation vs high FiO_2_ [10]	Targeting moderate saturations standard practice; reduces severe ROP without increasing mortality significantly	Standard of care; balancing risks is key [105]
Optimal nutrition	Provide substrate for growth and repair	Essential for normal lung development	Associated with better outcomes; aggressive nutrition standard practice	Standard of care; crucial for lung health [10]
Infection prevention	Reduce exposure to PAMPs and associated inflammation	Reduces inflammatory stimuli	Reduces sepsis rates, indirectly impacting BPD risk	Standard of care; critical component [105]

### 5.2. Established/Commonly Used Therapies

#### 5.2.1. Corticosteroids (Glucocorticoids)

Systemic corticosteroids remain the most potent anti‐inflammatory intervention but require a careful balance between efficacy and safety. While dexamethasone effectively facilitates extubation and reduces BPD rates, its association with adverse neurodevelopmental outcomes (e.g., cerebral palsy) limits its use to high‐risk infants who remain ventilator‐dependent. In contrast, recent evidence highlights low‐dose hydrocortisone as a potentially safer alternative. The PREMILOC trial demonstrated that early low‐dose hydrocortisone improved survival without BPD and, crucially, was not associated with the adverse neurodevelopmental effects seen with systemic dexamethasone.

#### 5.2.2. Caffeine

Beyond its role as a respiratory stimulant, caffeine exerts significant anti‐inflammatory and immunomodulatory effects. The landmark Caffeine for Apnea of Prematurity (CAP) trial provided Level I evidence that early caffeine therapy reduces the rate of BPD and improves long‐term neurodevelopmental outcomes at 18–21 months [106].

#### 5.2.3. Vitamin A

Intramuscular vitamin A supplementation modestly reduces BPD risk by supporting epithelial differentiation and repair. However, its clinical adoption remains limited due to the invasiveness of repeated intramuscular injections.

### 5.3. Investigational and Emerging Therapies

#### 5.3.1. Antioxidants (Beyond Vit A)

Despite the clear role of OS in BPD, clinical results for antioxidants have been mixed. Notably, while recombinant human CuZn superoxide dismutase (SOD) failed to reduce BPD rates at 36 weeks PMA in initial trials, long‐term follow‐up revealed a significant reduction in respiratory morbidity at 1 year of age [107]. This discrepancy underscores the importance of long‐term assessment in BPD trials, as structural benefits may manifest functionally later in life (Figure 4).

Figure 4Current and emerging therapeutic strategies targeting inflammation in bronchopulmonary dysplasia. Therapeutic interventions are categorized by their developmental status and mechanism of action. (A) Established therapies: Corticosteroids (e.g., Dexamethasone, Hydrocortisone) broadly suppress inflammation, while Caffeine antagonizes inflammation and stimulates respiratory drive. (B) Investigational anti‐inflammatory agents: These target specific molecular nodes, including IL‐1Ra (blocking IL‐1β), NLRP3 inflammasome inhibitors, PPAR‐γ agonists (inhibiting NF‐κB), and specific Antioxidants/KYC (scavenging ROS). (C) Regenerative approaches: Mesenchymal stem/stromal cells (MSCs) and their exosomes exert paracrine effects that inhibit inflammation and simultaneously promote angiogenesis (VEGF) and alveolar repair. (D) Supportive non‐pharmacological strategies: These preventive measures aim to block the initial triggers, including gentle ventilation (minimizing mechanical stress), optimized oxygen management (reducing hyperoxia), and optimal nutrition/infection control (reducing inflammatory stimuli and providing growth substrates).

**Figure:**
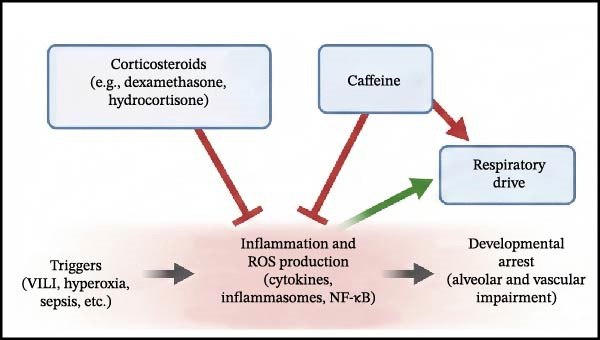
(A)

**Figure:**
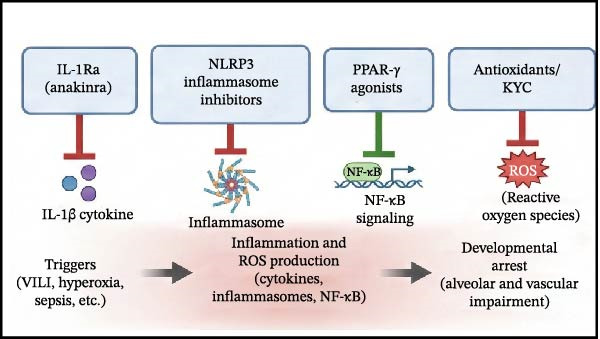
(B)

**Figure:**
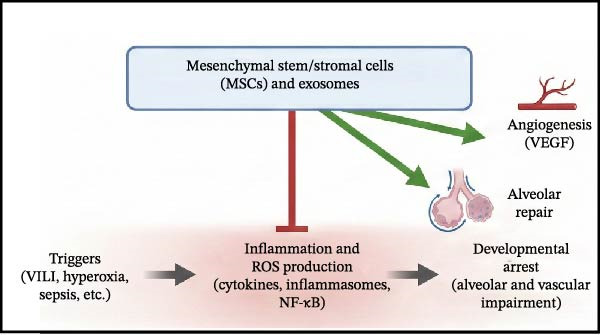
(C)

**Figure:**
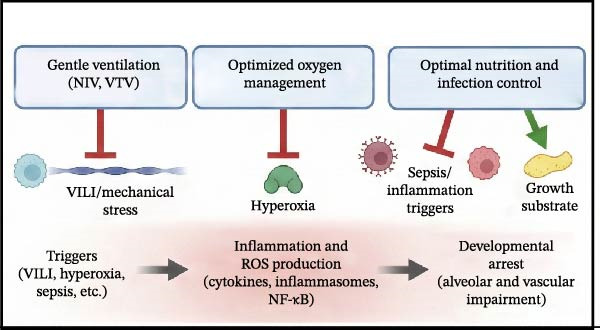
(D)

#### 5.3.2. PPAR‐γ Agonists

PPAR‐γ agonists (e.g., pioglitazone) have shown robust efficacy in preclinical models by blocking the NF‐κB pathway and preserving Wnt/VEGF signaling. Current research has moved towards clinical translation, with Phase I trials now underway to establish safe dosing regimens and pharmacokinetics in the preterm population.

#### 5.3.3. Stem Cell Therapy

Mesenchymal stem/stromal cells (MSCs) represent a promising frontier, exerting therapeutic effects primarily through a paracrine mechanism via the secretion of anti‐inflammatory and pro‐angiogenic factors. Recent pivotal work has further elucidated the therapeutic potential of MSC‐derived exosomes (extracellular vesicles) as a cell‐free alternative, showing efficacy in mitigating lung injury and promoting regeneration in preclinical models [108]. Early‐phase human trials affirm the safety of MSC administration, with larger efficacy trials currently in progress.

#### 5.3.4. Supportive Non‐Pharmacological Strategies

Optimizing the care environment is fundamental to minimizing inflammatory triggers. This includes “gentle ventilation” strategies (prioritizing non‐invasive support and volume‐targeted ventilation) to prevent VILI, and strict oxygen stewardship to avoid hyperoxia‐induced ROS generation. Furthermore, meticulous infection control and optimal nutrition are essential to limit systemic inflammation and provide the substrates necessary for lung repair.

## 6. Conclusion and Future Directions

### 6.1. Synthesis: Inflammation as a Linchpin in BPD

The collective body of evidence reviewed herein strongly reinforces the conclusion that inflammation serves as a central, unifying mechanism in the complex pathogenesis of bronchopulmonary dysplasia [64]. It is the linchpin that connects a diverse array of prenatal and postnatal insults—including infection, mechanical stress, and oxygen toxicity—to the arrested lung development seen in extremely preterm infants. This inflammatory response is often dysregulated, becoming exaggerated and persistent instead of resolving appropriately. This chronic inflammation, mediated by specific immune cells like neutrophils and macrophages and a complex network of cytokines, chemokines, ROS, and proteases, does not merely cause acute tissue damage [4]. Critically, its impact lies in the active disruption of fundamental molecular signaling pathways (such as VEGF, Wnt, and TGF‐β) that are indispensable for orchestrating normal alveolar septation and pulmonary angiogenesis during the saccular stage of lung development. This interference with developmental programing is the ultimate mechanism by which inflammation produces the hallmark pathologies of BPD: alveolar simplification and vascular dysgenesis.

### 6.2. Challenges and Unmet Needs

Despite significant progress in understanding the pathophysiology of BPD, particularly the role of inflammation, the clinical burden of the disease remains substantial [8]. The incidence of BPD, especially among the most extremely premature infants (<28 weeks gestation), has not significantly decreased over the past two decades and may even be increasing in some cohorts due to improved survival at lower gestational ages. This underscores a critical unmet need for more effective preventive and therapeutic strategies.

A major challenge lies in translating the promising findings from numerous preclinical studies, often conducted in animal models, into safe and effective therapies for human infants [13]. The disconnect between preclinical success and clinical failure stems from several factors. First, many animal models rely on acute injury (e.g., hyperoxia) in relatively mature lungs, which fails to capture the chronic, multifactorial nature of BPD in extremely preterm infants [109]. Second, the timing of intervention in humans is often reactive rather than preventive [110]. Third, BPD heterogeneity means that a therapy targeting a specific inflammatory pathway may only benefit a subset of infants with that specific endotype, yet clinical trials typically enroll “all‐comers,” diluting potential efficacy signals [111].

Many interventions that showed benefit in animals have failed to demonstrate efficacy or have revealed unacceptable side effects in clinical trials. Furthermore, BPD is recognized as a heterogeneous condition, likely encompassing different clinical phenotypes or endotypes driven by varying contributions of genetic factors, specific prenatal and postnatal insults, and differing underlying mechanisms (e.g., predominantly airway vs., parenchymal vs., vascular pathology) [112]. This heterogeneity complicates the development of “one‐size‐fits‐all” therapies and highlights the need for more personalized approaches.

### 6.3. Future Research Directions

Addressing these challenges requires continued, multifaceted research efforts. Key future directions include:

#### 6.3.1. Biomarkers and Early Prediction

There is a pressing need to develop and validate reliable biomarkers—potentially integrating inflammatory markers, genetic susceptibility profiles, metabolomic signatures, or imaging parameters—that can accurately identify infants at the highest risk of developing BPD very early in their postnatal course. Early risk stratification would enable the targeted application of potentially beneficial but perhaps risky preventive therapies only to those most likely to benefit.

#### 6.3.2. Phenotyping and Personalized Medicine

Moving beyond a single definition of BPD toward characterizing distinct clinical or pathophysiological subtypes (endotypes) is crucial [13]. Understanding whether an infant’s BPD is primarily driven by airway inflammation, alveolar simplification, or vascular disease could allow for more tailored therapeutic strategies.

#### 6.3.3. Novel Therapeutic Targets and Strategies

Continued basic and translational research is needed to identify and validate novel therapeutic targets within the inflammatory and developmental pathways. This includes further exploration of specific cytokine blockade (e.g., IL‐1), inflammasome inhibition, TLR modulation, targeting cellular senescence, enhancing endogenous anti‐inflammatory mechanisms (e.g., CC10, SP‐D), and refining regenerative approaches like MSC therapy [113, 114].

#### 6.3.4. Combination Therapies

Given the multifactorial nature of BPD, combination therapies targeting multiple pathways simultaneously (e.g., combining an anti‐inflammatory agent with an antioxidant or a pro‐angiogenic factor) may be more effective than single‐agent approaches and should be explored in preclinical models and potentially clinical trials [10].

#### 6.3.5. Long‐Term Outcomes Assessment

It is imperative that clinical trials evaluating new interventions for BPD include comprehensive and sufficiently long‐term follow‐up to assess not only short‐term respiratory outcomes but also crucial long‐term outcomes, including detailed neurodevelopmental assessments, pulmonary function trajectories into childhood, and cardiovascular health. Robust, multicenter, longitudinal studies are essential to truly understand the long‐term impact of interventions [115, 116].

In summary, inflammation stands as a pivotal mediator in the pathogenesis of BPD, linking multiple perinatal insults to the consequences of arrested lung development. While significant strides have been made in understanding these mechanisms, translating this knowledge into safe and effective therapies remains a major challenge. Future research focused on early risk prediction, personalized medicine, novel anti‐inflammatory and regenerative strategies, and the rigorous assessment of long‐term outcomes is essential to reduce the burden of this persistent neonatal lung disease.

**Nomenclature**
ASC: Apoptosis‐associated speck‐like protein containing a CARD
ATP: Adenosine triphosphate
BPD: Bronchopulmonary dysplasia
Casp‐1: Caspase‐1
COX‐2: Cyclooxygenase‐2
CXCL1: C‐X‐C motif chemokine ligand 1
CXCL8: C‐X‐C motif chemokine ligand 8 (also known as IL‐8)
CXCR2: C‐X‐C chemokine receptor type 2
DAMP: Damage‐associated molecular pattern
DAMPs: Damage‐associated molecular patterns
ECM: Extracellular matrix
ELGAN: Extremely low gestational age newborn
G‐CSF: Granulocyte colony‐stimulating factor
GSDMD: Gasdermin D
IFN‐γ: Interferon‐gamma
IL: Interleukin
IL‐1β: Interleukin‐1 beta
IL‐6: Interleukin‐6
iNOS: Inducible nitric oxide synthase
iPSC: Induced pluripotent stem cell
iPSC‐MSC: Induced pluripotent stem cell‐derived mesenchymal stem cell
LPS: Lipopolysaccharide
MEx: Mesenchymal stem cell‐derived exosomes
MSCs: Mesenchymal stem cells
NF‐κB: Nuclear factor kappa‐light‐chain‐enhancer of activated B cells
NLRP3: NOD‐like receptor family pyrin domain containing 3
NLRs: NOD‐like receptors
NO: Nitric oxide
PAMP: Pathogen‐associated molecular pattern
PGE2: Prostaglandin E2
RAGE: Receptor for advanced glycation end products
ROS: Reactive oxygen species
sVEGFR‐1: Soluble vascular endothelial growth factor receptor‐1
TGF‐β: Transforming growth factor‐beta
TLR4: Toll‐like receptor 4
TNF‐α: Tumor necrosis factor‐alpha
VEGF: Vascular endothelial growth factor.

## Author Contributions

Fei Wangconceptualized the study, collected and organized the literature, prepared the original draft, and contributed to its revision and finalization. Heng Zhang and Ou Jiang conducted the literature search, prepared the figures, and managed the references. Heng Zhang also performed proofreading. Hongying Mi designed the study, supervised the research process, reviewed the manuscript critically, and fulfilled the responsibilities of the corresponding author.

## Funding

This research was supported by the Intramural Fund of Faculty of Medicine of Kunming University of Science and Technology (KmustYxy‐2023015) and the Hospital Research Fund of Yunnan First People’s Hospital (YN2024‐017).

## Disclosure

All authors read and approved the final manuscript.

## Ethics Statement

The authors have nothing to report.

## Consent

The authors have nothing to report.

## Conflicts of Interest

The authors declare no conflicts of interest.

## Data Availability

Data sharing is not applicable to this article as no datasets were generated or analyzed during the current study.
